# Effect of motor process-related priming via repeated transcranial magnetic stimulation on embodiment perception during mirror visual feedback: a pilot study

**DOI:** 10.3389/fnins.2024.1501169

**Published:** 2024-11-26

**Authors:** Ying Li, Ping Li, Yixuan Li, Jia Wang, Xinyao Shen, Mingyong Zhang, Li Ding

**Affiliations:** ^1^Rehabilitation Department, Luqiao Hospital of Traditional Chinese Medicine, Tazihou, Zhejiang, China; ^2^Department of Rehabilitation Medicine, Huashan Hospital, Fudan University, Shanghai, China

**Keywords:** mirror visual feedback, embodiment perception, transcranial magnetic stimulation, motor process, dorsolateral prefrontal cortex

## Abstract

**Introduction:**

Non-invasive brain stimulation has been combined with mirror visual feedback (MVF) as a priming strategy to enhance therapeutic efficacy. However, a superior combined effect is hindered by the lack of emphasis on MVF-relevant embodiment perception.

**Objective:**

This study assessed the priming effect of repeated transcranial magnetic stimulation (TMS) over the primary motor cortex (M1) and dorsolateral prefrontal cortex (dlPFC) on embodiment perception during MVF.

**Methods:**

In the experiment, 15 healthy participants were required to complete tasks using their left hand while keeping their right hand static behind a mirror. They first received excitatory TMS over the left M1 or dlPFC, or sham-TMS in random order during three trial rounds and then performed three subsequent motor tasks and two task-oriented evaluations during MVF in each trial. Latency time (LT), number of embodiment occurrences, embodiment questionnaire (EQ) score, and time required to complete the task-oriented activities were recorded.

**Results:**

The results showed that the LT of forearm rotation in the dlPFC-TMS round was shorter than that in the sham-TMS round, although a greater number of occurrences were obtained in both the M1-TMS and dlPFC-TMS rounds compared to the sham-TMS round within the three motor tasks, which suggested that TMS priming facilitated the elicitation of embodiment perception. The EQ results indicated strengthened embodiment perception after TMS priming, especially in the dlPFC-TMS round.

**Conclusion:**

This study provides evidence that TMS priming over motor process-related regions, specifically the dlPFC, contributes to eliciting and intensifying embodiment perception during MVF, which benefited from a superior MVF paradigm for improving rehabilitation outcomes.

**Clinical Trial Registration:**

Identifier ChiCTR2400089499 https://www.chictr.org.cn/showproj.html?proj=240385.

## 1 Introduction

Mirror visual feedback (MVF) is an approved treatment for relieving pain and improving hand and upper limb functions and has been widely used in stroke rehabilitation (Thieme et al., 2018; Zhang et al., 2024; Wen et al., 2022; Guemann et al., 2023). During MVF, a plane mirror is placed midsagittally between the upper limbs, which reflects the movement of the unaffected limb to the affected side and prompts the illusion of two limbs moving synchronously in the participants. This optical illusion induced by MVF is relevant for the embodiment of mirrored reflection, which can determine the therapeutic efficacy of MVF (Mehnert et al., 2013; Longo et al., 2008).

Embodiment is a subjective experience that comprises a sense of location, ownership, agency, and deafference, of which perception relies on the integration of multisensory inputs (Longo et al., 2008; Azañón et al., 2016). During MVF, the visual input is the main origin of embodiment perception, and the more natural the mirrored reflection, the stronger the perceived degree (Wittkopf et al., 2017). In addition, bilateral and tool-based motor tasks are clinically used to facilitate embodiment perception during MVF, involving kinesthetic and tactile inputs. In our previous studies, vibrotactile stimulation and enunciations with auditory feedback were combined in separate MVF trainings for the hand and face, which augmented embodiment perception in healthy populations and those with facial palsy (Ding et al., 2020b; Ding et al., 2020a; Ding et al., 2023). Moreover, embodiment perception enhancement is accompanied by more obvious cortical excitability and efficient neural communication in healthy participants, which may benefit patients in stroke rehabilitation (Ding et al., 2020a, Ding et al., 2023). However, these approaches to increasing embodiment perception rely on a participant’s residual ability and are not applicable to stroke patients with severe sensorimotor dysfunction who are unable to perform bilateral movements or receive sensory inputs.

Non-invasive methods of brain stimulation, such as transcranial magnetic stimulation (TMS) and transcranial direct current stimulation (tDCS), are widely used for priming, providing a more plastic brain response for subsequent treatment (da Silva et al., 2020). Previous studies have reported a synergistic effect of one-session tDCS over the primary motor cortex (M1) before MVF for improving motor performance in healthy participants (von Rein et al., 2015; Hoff et al., 2015). Zhang and Fong (2019) suggested that repeated TMS over M1, in the form of intermittent theta burst stimulation, can upregulate the receptiveness of healthy participants’ brains to subsequent MVF, where a more obvious MVF-induced shift in sensorimotor event-related desynchronization can be obtained. In addition, a recent meta-analysis reported that the combined treatment of repeated TMS/tDCS over the motor cortex and MVF is more effective than a single treatment for stroke recovery, from the perspective of motor function, activity levels, and cortical excitability (Zhao et al., 2022). Conversely, Abdelhaleem et al. (2024) reported no additive effect of combining TMS/tDCS with MVF for sensorimotor function of the upper limb and cortical activation. Despite these positive results, no attention has been paid to the priming effect of non-invasive brain stimulation on embodiment perception during MVF, which might be one possible explanation for the inconsistent results and hinder further exploration of the priming effect (Abdelhaleem et al., 2024).

Repeated TMS and tDCS have been applied to motor-related areas in previous studies, particularly M1, which is important in motor execution and is modulated by MVF for motor relearning (Zhao et al., 2022; Deconinck et al., 2015). In addition, as visually guided motor imagery, the influence of MVF on upstream motor processes, including planning and preparation, has been reported, which might be recognized as a potential target of neural modulation (Deconinck et al., 2015; Ding et al., 2018; Cheng et al., 2019; Guo et al., 2016; Polli et al., 2017; Falbo et al., 2024). The dorsolateral prefrontal cortex (dlPFC) is a higher-order region involved in executive functions, including visuospatial working memory and planning, whose activity is also modulated during MVF-induced intermodal conflict (Webler et al., 2022; Barbey et al., 2013; Lin et al., 2021; Deconinck et al., 2015). Thus, we hypothesized that priming over motor process-related regions, emphasizing the M1 (execution) and dlPFC (preparation), might benefit embodiment perception during MVF.

In the present study, repeated TMS was used as suggested by Zhao et al. (2022) to have a more significant additive effect with MVF. The aim of the study was to investigate the priming effect of repeated TMS over the M1 and dlPFC on embodiment perception during MVF separately via behavioral assessments and questionnaire surveys in healthy populations, compared to those without priming (sham-TMS). This pilot study will contribute to presenting an optimal priming strategy for repeated TMS combined with MVF and provide new insights for augmenting outcomes in stroke rehabilitation.

## 2 Materials and methods

### 2.1 Study design and participants

**Figure 1:**
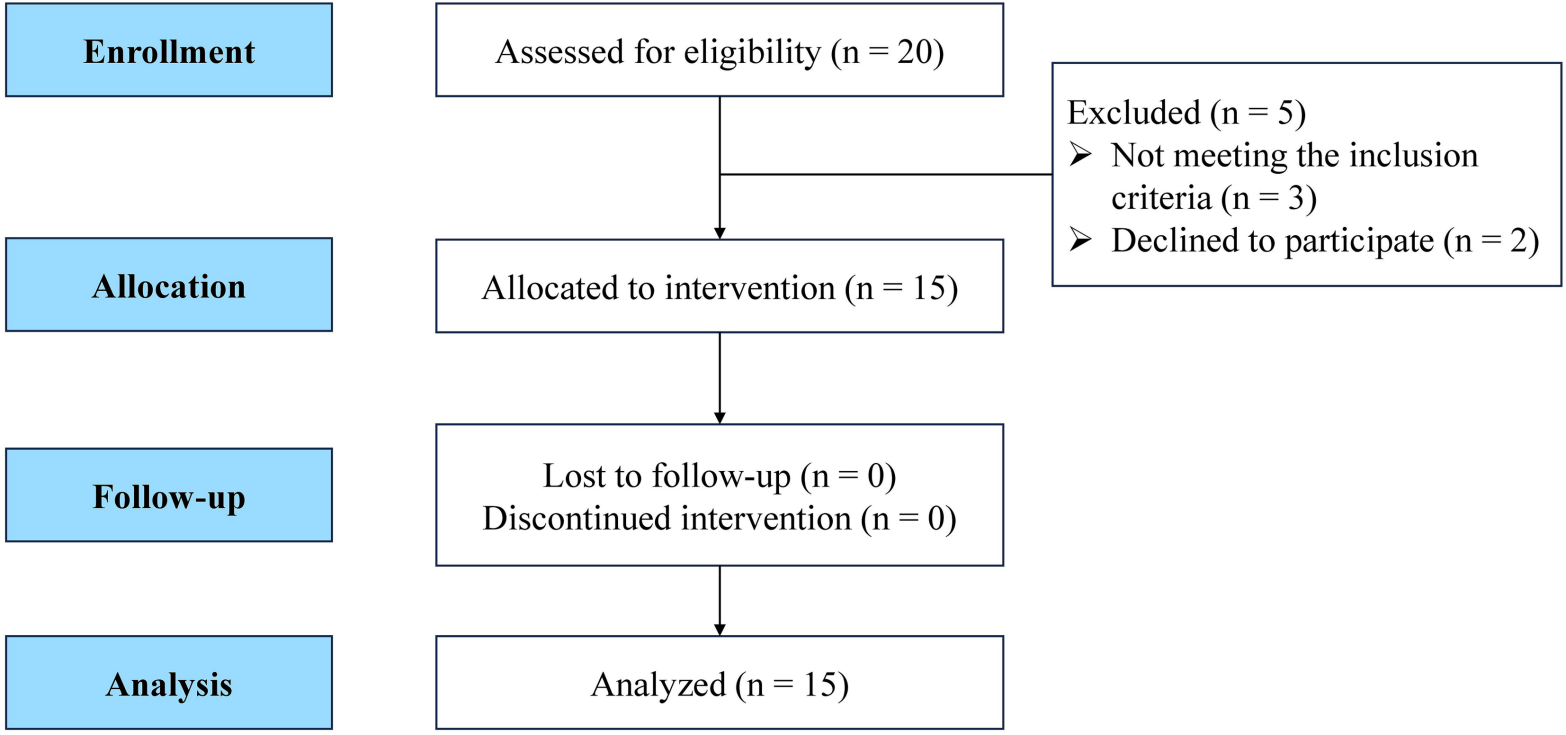
STROBE flow diagram of the study design.

This randomized, observational crossover study used a within-participant design trial to compare the effects of repeated TMS priming over motor process-related regions on embodiment perception during MVF. A total of 15 healthy right-handed participants were enrolled in the experiment (mean age: 24.53 ± 3.93 years; 7 women and 8 men; mean BMI: 21.56 ± 3.81 kg/m^2^) (see Figure 1). None of the participants had previously participated in any MVF studies or experiments.

All participants signed informed consent forms prior to the experiment, which was approved by the Luqiao Hospital of Traditional Chinese Medicine Institutional Review Boards prior to enrollment. The participants whose photographs are shown in the present study provided written informed consent for photo/video releases. This study was registered in the Chinese Clinical Trial Registry (ChiCTR2400089499).

### 2.2 Experimental protocol

**Figure 2:**
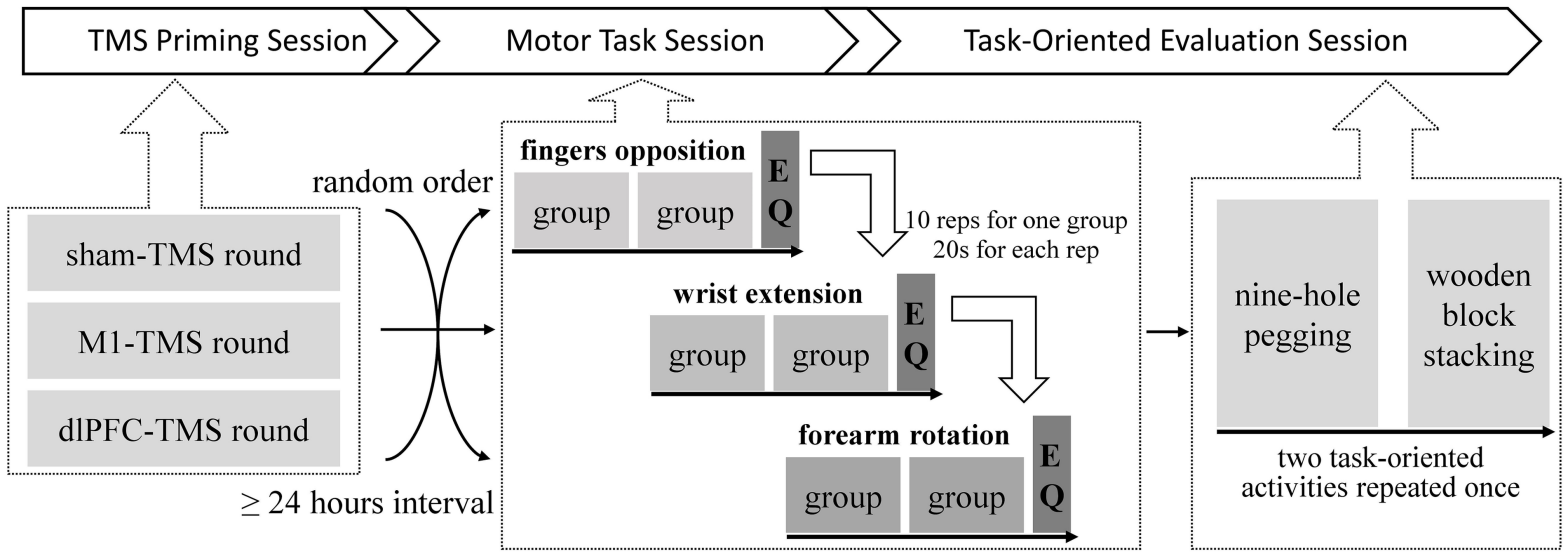
Demonstration of the experimental protocol.

In this experiment, all participants underwent three rounds of trials containing three sessions sequentially: TMS priming, motor tasks, and task-oriented evaluation under MVF (Figure 2). The following three conditions of repeated TMS priming were included in this study: priming over the M1 (M1-TMS), priming over the dlPFC (dlPFC-TMS), and placebo priming (sham-TMS). Each round included a specific condition of repeated TMS priming, and the three rounds of trials were randomly ordered with an interval of more than 24 h.

#### 2.2.1 Repeated TMS priming session

In the priming session, the YRD CCY-I Magnetic Stimulator (Yiruide Co. Ltd., Wuhan, China) with an “8”-shaped coil rotated at a 45° angle off the midline was used to provide repeated TMS. The resting motor threshold (RMT) was measured for each participant and was defined as the minimum intensity over the hotspot of the left M1 (representing right hand, as all participants were right-handed), which elicited a motor evoked potential of no less than 50 μV in the first dorsal interosseus muscle of the right hand in five out of 10 trials. A hotspot was defined as the coil position that elicited the largest motor-evoked potential.

The priming protocol was proposed according to previous studies and the results of our preliminary experiment (Kim and Yim, 2018; Tik et al., 2017; Avissar et al., 2017) in which 30 trains of stimulation with a frequency of 10 Hz were applied at sites of the left M1 or the left dlPFC with an intensity equal to 90% RMT. Each train duration was 5 s with an interval of 25 s, and the priming session contained 1,500 pluses within 15 min. In the M1-TMS round, the stimulation site was set at the hotspot of the left M1. In the dlPFC-TMS round, the stimulation site was set using the Beam F3 system (Beam et al., 2009). In the sham-TMS round, the stimulation site was set over the vertex in only 20% of the individual RMTs.

#### 2.2.2 Motor task session

In the motor task session, a plane mirror (40 cm × 60 cm) was used to provide the MVF and was sagittally positioned in the middle of two hands, which were 30 cm apart. As all participants were right-handed, the left limb was active in providing reflection, and the right limb was placed at the back of the mirror. To avoid the distraction of visual input from the left side, a shield was used and set above the left limb (Supplementary Figure S1).

The participants were asked to perform three motor tasks under MVF after TMS/sham-TMS priming using their left hand, including finger opposition (four fingers touching the thumb and repositioning), wrist extension, and forearm rotation (pronation and supination) (Supplementary Video S1). Each motor task was repeated 10 times for the two groups. In each repetition, participants were required to conduct the motor task at a moderate speed for 20 s, keep the right limb static, gaze at the mirror, persuade themselves that their left limbs were moving, and tell the researcher when there was a sense of embodiment perception. The instructions for the sense were “the hand in the mirror was my right hand” and “my right hand was moving.” Breaks were set between repetitions (5 s), groups (30 s), and motor tasks (2 min), and an embodiment questionnaire (EQ) was administered after each motor task (see 2.3.2 EQ survey).

#### 2.2.3 Task-oriented evaluation session

In the task-oriented evaluation session, we aimed to objectively measure embodiment perception via the following two task-oriented activities: nine-hole pegging and wooden block stacking (Supplementary Video S1). The same mirror setting as in the motor task session was used, and the participants were required to gaze at the mirror and keep their right hand static. Due to the obstructed view from the left side, the participants had to rely on mirrored reflection as visual guidance and use their left hand to complete task-oriented activities. Thus, we proposed that better performance of task-oriented activities suggested a stronger embodiment perception of the mirrored reflection, and the motor control of the left hand was dependent on the sense of location, ownership, and agency from the mirrored reflection (Longo et al., 2008).

In the nine-hole pegging test, the participants took the pegs individually using their left hand and placed them into the holes on the wooden board, which was set 15 cm apart from the mirror, as quickly as possible. Peg placement sequencing was not required, and the time required to complete the activity was recorded. For wooden block stacking, participants were asked to stack five wooden blocks (2 cm × 2 cm), which were placed in a line 15 cm apart from the mirror, using their left hand. The blocks had to be successfully and steadily stacked, and the time required to complete the activity was recorded. Other requirements were the same as those in the motor task session; however, the participants only performed the two activities once separately, and the EQ was not administered. Practices were allowed for a better understanding of the processes and requirements.

### 2.3 Outcome measurements

#### 2.3.1 Latency time and number of embodiment occurrences

Latency time (LT) was defined as the time from the onset of each motor task repetition to embodiment perception. The maximum LT was 20 s for those who had no embodiment perception in a repetition, and the average LT for 20 repetitions (10 repetitions/group × 2 groups) was calculated for comparison within three rounds. In addition, the number of embodiment occurrences was defined as the number of successful embodiment perception occurrences for each motor task, with 10 being the maximum value for each group. The average number of occurrences for the two groups for each motor task was used for comparison within the three rounds. The shorter the LT and the greater the number of embodiment occurrences, the more positive the effect of TMS priming on the elicitation of embodiment perception during MVF.

#### 2.3.2 EQ survey

An EQ was administered after each motor task in the three rounds. The EQ evaluated the embodiment perception from four aspects, including symmetry (S), ownership (O), agency (A), and deafference (D), which consisted of eight statements with scores ranging from −5 to 5. The eight statements contained symmetry of reflection (S1, “the hand in the mirror seemed natural,” S2, “the hand in the mirror overlapped with my right hand behind the mirror”), ownership of the reflection (O1, “It felt like I was looking directly at my right hand rather than at a reflection of the hand,” O2, “It seemed the right hand in the mirror was part of my body”), agency of the reflection (A1, “It felt like that my right hand was moving with the reflection in the mirror,” A2, “It seemed I could control the movement of the hand in the mirror without moving my left hand”), and deafference (D-1 “It felt like I could not tell where my right hand was,” D-2 “My right hand felt unusual”). Higher scores indicated stronger agreement for each statement and suggested a more distinct sense of embodiment perception. The EQ was used to investigate the effect of TMS priming on the degree of embodiment perception during MVF, as was used in our previous studies (Ding et al., 2020b; Ding et al., 2020a).

#### 2.3.3 Time taken for task-oriented activity

The time taken to successfully complete the two task-oriented activities was used to objectively investigate the effect of TMS priming on enhancing the degree of embodiment perception during MVF. As clarified in the task-oriented evaluation session, better performance was associated with a stronger embodiment perception. Thus, a shorter time required to complete the activities indicated a stronger degree of embodiment perception.

### 2.4 Analyses

The Shapiro–Wilk test was used for checking distribution normality, and Levene’s test was used for the homogeneity of variances. The values of the average LT for the three motor tasks were compared within the three rounds separately using a one-way analysis of variance (ANOVA). The three rounds of TMS priming (M1-TMS, dlPFC-TMS, and sham-TMS) were the three levels of a single factor, and the participant was a random factor. The average number of embodiment occurrences and the EQ for the three motor tasks were compared in three rounds using the Friedman test. For significant data, *post-hoc* analyses were conducted using the Wilcoxon signed-rank test. The time taken for the two task-oriented activities was compared within three rounds using the Friedman test and further analyzed using the Wilcoxon signed-rank test.

Statistical analyses were performed using SPSS 24 (IBM Corp., Armonk, NY, United States), and the significance level was set at 0.05 using a two-sided test.

## 3 Results

### 3.1 Latency time for motor tasks

The results of the one-way ANOVA for LT are presented in Table 1. A significant difference was observed in the forearm rotation motor task among the three rounds (F_2,42_ = 3.482, *p* = 0.04). The *post-hoc* analysis showed that the LT of forearm rotation in the dlPFC-TMS round was shorter than that in the sham-TMS round (*p* = 0.012), which indicated the capability of dlPFC-TMS priming to facilitate the elicitation of embodiment perception. However, no significant differences were observed in other rounds or motor tasks.

**Table 1:** Results of latency time for motor tasks and time taken for task-oriented activities within 3 rounds of TMS priming.

	Sham-TMS (*n* = 15)	M1-TMS (*n* = 15)	dlPFC-TMS (*n* = 15)	*F*	*p*
Motor task (latency time, mean ± sd)					
Fingers opposition	15.83 ± 3.02	14.11 ± 4.45	13.40 ± 4.17	1.513	0.232
Wrist extension	15.87 ± 3.22	15.09 ± 4.06	13.96 ± 3.76	1.016	0.371
Forearm rotation	17.03 ± 2.46	15.17 ± 3.69	13.68 ± 4.10	3.482	0.040*
Task-oriented activity (time, mean ± sd)					
Nine-hole pegging	55.89 ± 15.90	52.13 ± 16.18	41.05 ± 11.34	4.166	0.022*
Wooden block stacking	18.52 ± 7.39	15.74 ± 9.28	14.22 ± 7.93	1.050	0.359

### 3.2 Number of embodiment occurrences for motor tasks

The number of embodiment occurrences for all motor tasks had significant differences between the three rounds (Table 2). Further analyses demonstrated that the number of embodiment occurrences was significantly greater in the M1-TMS and the dlPFC-TMS rounds than in the sham-TMS round for three motor tasks (finger opposition: M1-TMS vs. sham-TMS, *p* = 0.016, dlPFC-TMS vs. sham-TMS, *p* = 0.012; wrist extension: M1-TMS vs. sham-TMS, *p* = 0.01, dlPFC-TMS vs. sham-TMS, *p* = 0.025; forearm rotation: M1-TMS vs. sham-TMS, *p* = 0.002, dlPFC-TMS vs. sham-TMS, *p* = 0.002), which indicated that TMS priming over M1 or dlPFC had the capability in inducing embodiment perception. No significant differences in the number of embodiment occurrences were observed between the rounds of M1-TMS and dlPFC-TMS.

**Table 2:** Results of the number of embodiment occurrences for three motor tasks within three rounds of TMS priming.

	Sham-TMS (*n* = 15)	M1-TMS (*n* = 15)	dlPFC-TMS (*n* = 15)	X^2^	*p*
Numbers of embodiment, median [range]					
Fingers opposition	7.33 [0.00, 9.67]	8.00 [1.67, 10.00]	8.33 [2.33, 10.00]	14.250	0.001*
Wrist extension	5.67 [0.00, 10.00]	7.33 [0.33, 10.00]	8.33 [2.00, 10.00]	8.179	0.017*
Forearm rotation	6.67 [0.00, 9.67]	7.67 [0.00, 10.00]	8.33 [1.67, 10.00]	15.709	<0.001*

### 3.3 Time taken for task-oriented activities

The results of the one-way ANOVA on the time taken for the two task-oriented activities within the three rounds are presented in Table 1. A significant difference was observed only in the nine-hole pegging task (F_2, 42_ = 4.166, *p* = 0.022). The *post-hoc* analysis demonstrated that the time taken for nine-hole pegging in the dlPFC-TMS round was significantly shorter than that in the other two rounds (dlPFC-TMS vs. M1-TMS, *p* = 0.044; dlPFC-TMS vs. sham-TMS, *p* = 0.008), which suggested a stronger degree of embodiment perception during MVF after dlPFC-TMS priming.

### 3.4 EQ

**Figure 3:**
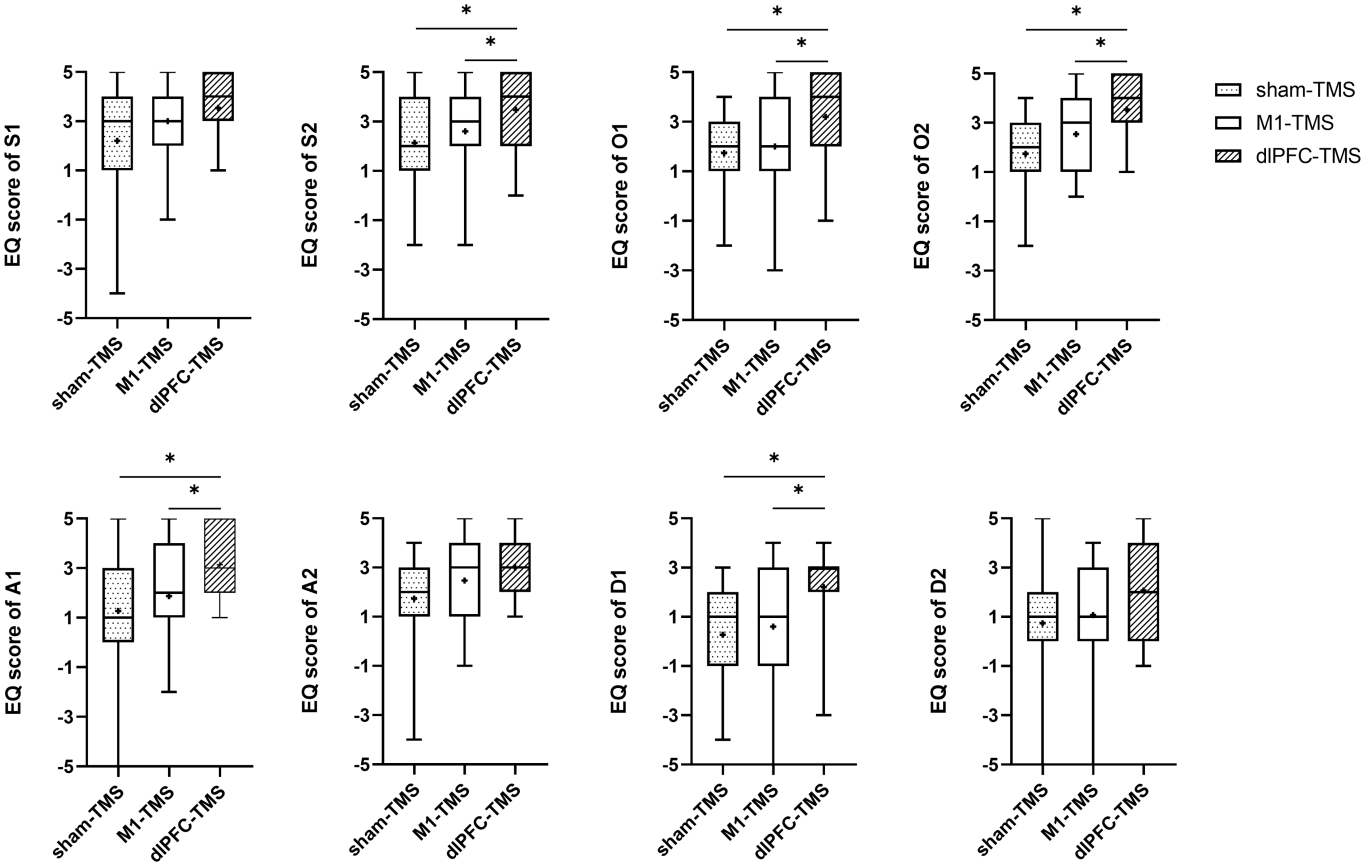
Results of the embodiment questionnaire (EQ) among three rounds of trial (sham-TMS, M1-TMS, and dlPFC-TMS) for the motor task of forearm rotation. *, *p* < 0.05.

**Figure 4:**
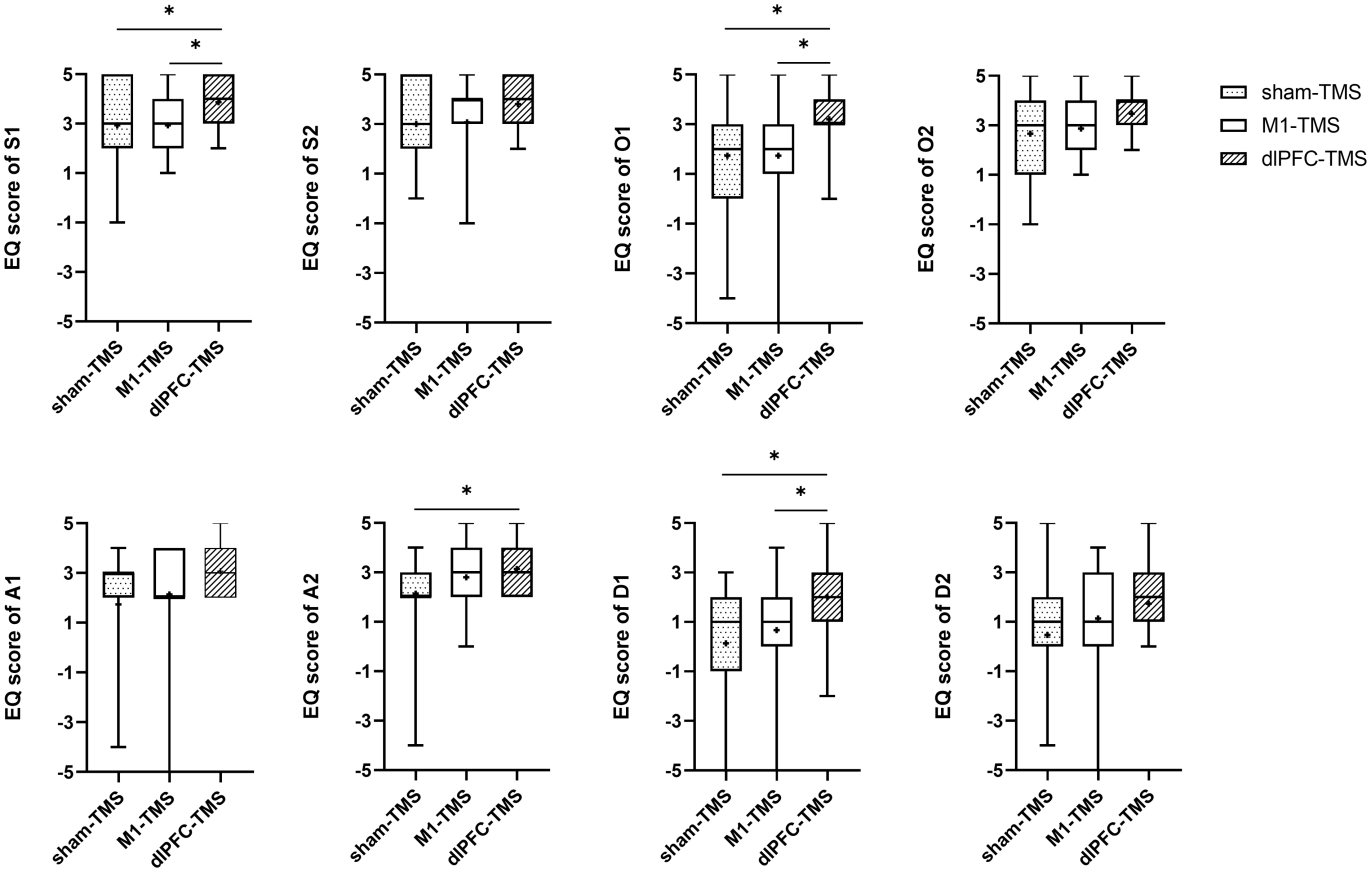
Results of the embodiment questionnaire (EQ) among three rounds of trial (sham-TMS, M1-TMS, and dlPFC-TMS) for the motor task of wrist extension. *, *p* < 0.05.

**Figure 5:**
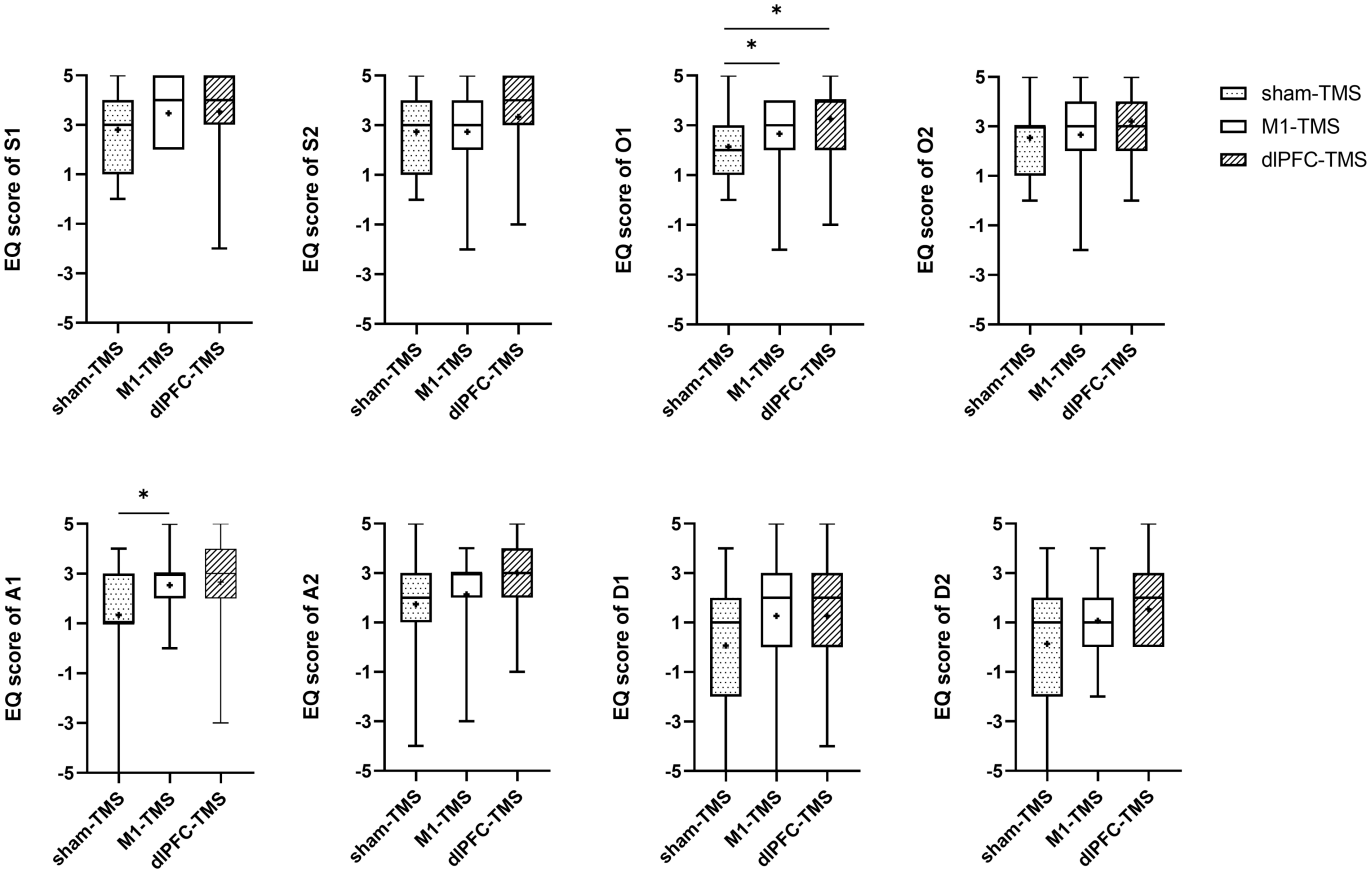
Results of the embodiment questionnaire (EQ) among three rounds of trial (sham-TMS, M1-TMS, and dlPFC-TMS) for the motor task of finger opposition. *, *p* < 0.05.

All participants reported experiences of embodiment during the three motor tasks under MVF and varied degrees of embodiment within three rounds. The results of the Friedman test showed significant agreement for half the statements on embodiment perception for the three motor tasks (Supplementary Table S1), and further analyses indicated stronger agreement for the dlPFC-TMS round, especially the motor task of forearm rotation (Figures 3–5; Supplementary Table S2).

For the forearm rotation motor task, participants more strongly agreed with the statements on symmetry, ownership, agency, and deafference for the dlPFC-TMS round than for the M1-TMS and sham-TMS rounds (S2, O1, O2, A1, and D-1, all *p* < 0.05); and participants more strongly agreed with the statements on ownership for the M1-TMS round than for the sham-TMS round (O2, *p* = 0.042) (Figure 3; Supplementary Table S2).

For the wrist extension motor task, participants more strongly agreed with the statements on symmetry, ownership, and deafference for the dlPFC-TMS round than for the other two rounds (S1, O1, and D1, all *p* < 0.05) (Figure 4; Supplementary Table S2); and participants more strongly agreed with the statements on agency for the dlPFC-TMS round than for the sham-TMS round (A2, *p* = 0.013).

For the finger opposition motor task, participants more strongly agreed with the statements on ownership for the dlPFC-TMS round (O1, *p* = 0.043) and the M1-TMS round (O1, *p* = 0.028) than for the sham-TMS round; and participants more strongly agreed with the statements on agency for the M1-TMS round than for the sham-TMS round (A1, *p* = 0.011).

## 4 Discussion

The present study provides behavioral evidence that priming over motor process-related regions via repeated TMS contributed to the elicitation and intensification of MVF-relevant embodiment perceptions in healthy participants. Moreover, a superior modulatory effect of dlPFC-TMS priming on embodiment perception during MVF was observed, which emphasized the upstream motor process compared to motor execution-associated M1-TMS priming.

### 4.1 Effect on the elicitation and intensity of embodiment perception

The combined treatment effect of non-invasive brain stimulation with MVF, specifically TMS and tDCS, has been investigated in recent studies in which non-invasive methods were used to prime over the M1 for a better response to subsequent MVF (von Rein et al., 2015; Hoff et al., 2015; Zhang and Fong, 2019; Zhao et al., 2022; Abdelhaleem et al., 2024). Although positive results have been obtained in healthy and stroke populations, few studies have emphasized the additive effect of priming on embodiment perception related to the MVF, which is an important factor for efficacy (Mehnert et al., 2013). In the present study, we found that the elicitation and intensification of embodiment perception were facilitated by TMS priming, especially dlPFC-TMS priming, as suggested by the results of the behavioral assessments and embodiment surveys from the healthy participants. Similar to our previous studies (Ding et al., 2020b; Ding et al., 2020a), shorter latency times and stronger agreement for statements in the EQ were obtained from participants with stronger embodiment perception. Task-oriented activities were used in this present study to objectively evaluate the intensity of embodiment perception. As clarified in the experimental protocol section, better performance corresponded to stronger embodiment perception; the shorter time required to complete the nine-hole pegging under MVF indicated the significant effect of dlPFC-TMS priming on strengthening embodiment perception, compared to the priming via M1-TMS and sham-TMS. Thus, our study extends previous findings regarding the effect of TMS priming strengthening embodiment perception during MVF and suggests that the dlPFC is an optimal stimulation position. Moreover, this finding may help explain the reasons for conflicting results of the combined effect on sensorimotor improvement and cortical excitability in previous studies that priming set over M1 with the limitation of distinctive embodiment perception (Zhao et al., 2022, Abdelhaleem et al., 2024).

### 4.2 Effect of motor process-related priming on embodiment perception

In the present study, we used excitatory repeated TMS to prime over motor process-involved regions, including the M1 and dlPFC, and a positive impact on MVF-induced embodiment perception was obtained. The MVF essentially transfers visual input to motor output. Embodiment perception plays a critical role in the transfer, which represents successful multisensory integration and is influenced by kinesthetic motor imagery in the top-down process (Bello et al., 2020; Chancel et al., 2017; Metral et al., 2015). As a part of high-order processes, kinesthetic motor imagery involves maintaining visual information and rehearsing movements in working memory and elicits motor execution (Hetu et al., 2013; Simos et al., 2017). Thus, priming over the M1 and dlPFC strengthens the role of kinesthetic motor imagery in MVF, which might have a positive effect on embodiment perception (Simos et al., 2017). In addition, many studies have reported an association between embodiment perception and activation of M1 and dlPFC during MVF (Deconinck et al., 2015; Qiu et al., 2022; Nojima et al., 2012). In our previous electroencephalogram (EEG) studies, we found strengthened embodiment perception via a combination of vibrotactile input accompanied by increased desynchronization and node degrees on channels C3 and F3 corresponded to M1 and dlPFC (Ding et al., 2020a; Ding et al., 2023; Beam et al., 2009; Silva et al., 2021). Another possible interpretation is that the lasting effect of repeated TMS on the decreased activation threshold of the M1 and dlPFC may contribute to a better response to MVF (Ziemann et al., 2008; Hayashi et al., 2004).

Furthermore, in our study, a prominent effect of dlPFC-TMS priming on embodiment perception was observed compared to that of M1-TMS and sham-TMS priming. MVF can influence motor preparation and planning according to the principles of graded motor imagery (Ding et al., 2018; Polli et al., 2017; Falbo et al., 2024; Hanakawa et al., 2008; Bowering et al., 2013). Bello et al. (2020) and Bello et al. (2021) demonstrated that top-down attention and working memory played critical roles in MVF. Thus, priming the dlPFC with visuospatial memory and attentional control may result in a superior effect on embodiment perception during MVF (Webler et al., 2022; Lee et al., 2020). Moreover, the dlPFC is the end point for the dorsal visual stream concerning visual field perception and awareness and is critical for the activation of the M1 during MVF, which suggests a hub role for the dlPFC in visuo-motor transformation in MVF (Nojima et al., 2012; Takahashi et al., 2013). This finding supports the importance of the dlPFC in embodiment perception during MVF.

Notably, we observed a limited effect of M1-TMS priming on embodiment perception. In addition to the significantly enhanced number of embodiment occurrences in the three motor tasks, only stronger agreement for O1 and A1 in finger opposition and O2 in forearm rotation was obtained in the M1-TMS round compared to the sham-TMS round. This may have resulted in the stimulation site being over the hotspot of the left M1 (hand representation), which only emphasized the motor execution of the hand with less impact on the entire MVF process. In contrast, Casula et al. (2022) reported decreased activation of the M1 while perceiving the embodiment of a virtual hand, which suggests that inhibitory TMS over the M1 might benefit embodiment perception in MVF. In addition, our study demonstrated a superior effect of TMS priming on the elicitation and intensity of embodiment perception during MVF for the motor task of forearm rotation. This finding suggested a potential interaction of motor process-related priming and motor tasks with limb rotation during the MVF paradigm, which involved extra mental demands of spatial cognition, and guided future studies and clinical applications in designing motor tasks.

### 4.3 Limitations

The present study has several limitations. First, we only measured behavioral data from task performance and questionnaires to evaluate embodiment perception, which compromised both the internal validity and the construct validity of the interpretations of our findings. Although task-oriented activities during MVF were applied to provide an objective evaluation, electrophysiological techniques or brain imaging methods, such as EEG or functional near-infrared spectroscopy, should be used in future studies to evaluate MVF-relevant alterations of cortical activation, which might contribute to providing more solid evidence and guide future studies in potential neuromechanism. Moreover, possible protective factors influencing the survey should be evaluated in future studies. Second, inhibitory TMS over left dlPFC and M1 or the contralateral sites should be used as controls to test the effect on embodiment perception during MVF, which could be of benefit to improve the interpretation of our findings. Third, we only emphasized the top-down motor processes of the MVF. Possible priming sites related to bottom-up neural processes, especially for perceptual input and sensorimotor integration, should be considered for further comparisons. Finally, future studies should recruit older populations or patients with phantom limb pain or hemiparesis to provide more information to reinforce the MVF paradigm and further explore the therapeutic efficacy of the novel paradigm on pain management and motor recovery.

## 5 Conclusion

The present study showed that repeated TMS priming over the M1 and dlPFC, which correspond to execution and planning/preparation in motor processes, respectively, had a beneficial effect of eliciting and intensifying embodiment perception during MVF in healthy participants. Furthermore, our study result suggests an optimal priming site over the dlPFC with a prominent effect on embodiment perception. These findings suggest the potential clinical implications of targeting the dlPFC in TMS combined with MVF, which reinforces the MVF-relevant rehabilitation protocol for improving outcomes in the management of phantom limb pain and neurorehabilitation after stroke.

## Data Availability

The raw data supporting the conclusions of this article will be made available by the authors, without undue reservation.
