# Supplementary material for: Effect of motor process-related priming via repeated transcranial magnetic stimulation on embodiment perception during mirror visual feedback: a pilot study
Source: Front Neurosci. 2024 Nov 26;18:1501169. doi: 10.3389/fnins.2024.1501169 (PMC11628548; doi:10.3389/fnins.2024.1501169)
Supplement: Supplementary file 1 [file Data_Sheet_1.DOCX]

**Supplementary**

**Table S1** The results of the Friedman test on embodiment questionnaire for three motor tasks within three rounds of TMS priming.

|  | | sham-TMS  median [range] | M1-TMS  median [range] | dlPFC-TMS  median [range] | X^2^ | *p* |
| --- | --- | --- | --- | --- | --- | --- |
| S-1 | finger opposition | 3.0 [0.0, 5.0] | 4.0 [2.0, 5.0] | 4.0 [-2.0, 5.0] | 4.512 | 0.105 |
|  | wrist extension | 3.0 [-1.0, 5.0] | 3.0 [1.0, 5.0] | 4.0 [2.0, 5.0] | 9.116 | 0.010* |
|  | forearm rotation | 3.0 [-4.0, 5.0] | 3.0 [-1.0, 5.0] | 4.0 [1.0, 5.0] | 5.905 | 0.052 |
| S-2 | finger opposition | 3.0 [0.0, 5.0] | 3.0 [-2.0, 5.0] | 4.0 [-1.0, 5.0] | 4.650 | 0.098 |
|  | wrist extension | 3.0 [0.0, 5.0] | 4.0 [-1.0, 5.0] | 4.0 [2.0, 5.0] | 3.682 | 0.159 |
|  | forearm rotation | 2.0 [-2.0, 5.0] | 3.0 [-2.0, 5.0] | 4.0 [0.0, 5.0] | 8.440 | 0.015* |
| O-1 | finger opposition | 2.0 [0.0, 5.0] | 3.0 [-2.0, 4.0] | 4.0 [-1.0, 5.0] | 11.551 | 0.003* |
|  | wrist extension | 2.0 [-4.0, 5.0] | 2.0 [-5.0, 5.0] | 3.0 [0.0, 5.0] | 6.980 | 0.031* |
|  | forearm rotation | 2.0 [-2.0, 4.0] | 2.0 [-3.0, 5.0] | 4.0 [-1.0, 5.0] | 11.818 | 0.003* |
| O-2 | finger opposition | 3.0 [0.0, 5.0] | 3.0 [-2.0, 5.0] | 3.0 [0.0, 5.0] | 4.545 | 0.103 |
|  | wrist extension | 3.0 [-1.0, 5.0] | 3.0 [1.0, 5.0] | 4.0 [2.0, 5.0] | 6.936 | 0.031* |
|  | forearm rotation | 2.0 [-2.0, 4.0] | 3.0 [0.0, 5.0] | 4.0 [1.0, 5.0] | 16.178 | <0.001* |
| A-1 | finger opposition | 1.0 [-5.0, 4.0] | 3.0 [0.0, 5.0] | 3.0 [-3.0, 5.0] | 12.943 | 0.002* |
|  | wrist extension | 3.0 [-4.0, 4.0] | 2.0 [-5.0, 4.0] | 3.0 [2.0, 5.0] | 5.773 | 0.056 |
|  | forearm rotation | 1.0 [-5.0, 5.0] | 2.0 [-2.0, 5.0] | 3.0 [1.0, 5.0] | 8.000 | 0.018* |
| A-2 | finger opposition | 2.0 [-4.0, 5.0] | 3.0 [-3.0, 4.0] | 3.0 [-1.0, 5.0] | 4.933 | 0.085 |
|  | wrist extension | 2.0 [-4.0, 4.0] | 3.0 [0.0, 5.0] | 3.0 [2.0, 5.0] | 7.737 | 0.021* |
|  | forearm rotation | 2.0 [-4.0, 4.0] | 3.0 [-1.0, 5.0] | 3.0 [1.0, 5.0] | 3.767 | 0.152 |
| D-1 | finger opposition | 1.0 [-5.0, 4.0] | 2.0 [-5.0, 5.0] | 2.0 [-4.0, 5.0] | 5.216 | 0.074 |
|  | wrist extension | 1.0 [-5.0, 3.0] | 1.0 [-5.0, 4.0] | 2.0 [-2.0, 5.0] | 8.415 | 0.015* |
|  | forearm rotation | 1.0 [-4.0, 3.0] | 1.0 [-5.0, 4.0] | 1.0 [-3.0, 4.0] | 10.939 | 0.004* |
| D-2 | finger opposition | 1.0 [-5.0, 4.0] | 1.0 [-2.0, 4.0] | 2.0 [0.0, 5.0] | 6.000 | <0.05* |
|  | wrist extension | 1.0 [-4.0, 5.0] | 1.0 [-5.0, 4.0] | 2.0 [0.0, 5.0] | 5.320 | 0.070 |
|  | forearm rotation | 1.0 [-5.0, 5.0] | 1.0 [-5.0, 4.0] | 2.0 [-1.0, 5.0] | 5.434 | 0.066 |

**Table S2** The *p*-values of pairwise comparison in embodiment questionnaire within three rounds of TMS priming. Only motor tasks with significant results of the Friedman test were presented.

|  | | M1-TMS vs. sham-TMS | dlPFC-TMS vs. sham-TMS | dlPFC-TMS vs. M1-TMS |
| --- | --- | --- | --- | --- |
| S-1 | wrist extension | 1.000 | 0.043* | 0.004* |
| S-2 | forearm rotation | 0.395 | 0.049* | 0.038* |
| O-1 | finger opposition | 0.028* | 0.043* | 0.179 |
|  | wrist extension | 0.873 | 0.036* | 0.016* |
|  | forearm rotation | 0.503 | 0.018* | 0.012* |
| O-2 | wrist extension | 0.586 | 0.054 | 0.084 |
|  | forearm rotation | 0.046* | 0.003* | 0.008* |
| A-1 | finger opposition | 0.011* | 0.079 | 0.227 |
|  | forearm rotation | 0.347 | 0.036* | 0.031* |
| A-2 | wrist extension | 0.121 | 0.013* | 0.212 |
| D-1 | wrist extension | 0.500 | 0.032* | 0.010* |
|  | forearm rotation | 0.403 | 0.012* | 0.011* |
| D-2 | finger opposition | 0.080 | 0.079 | 0.262 |


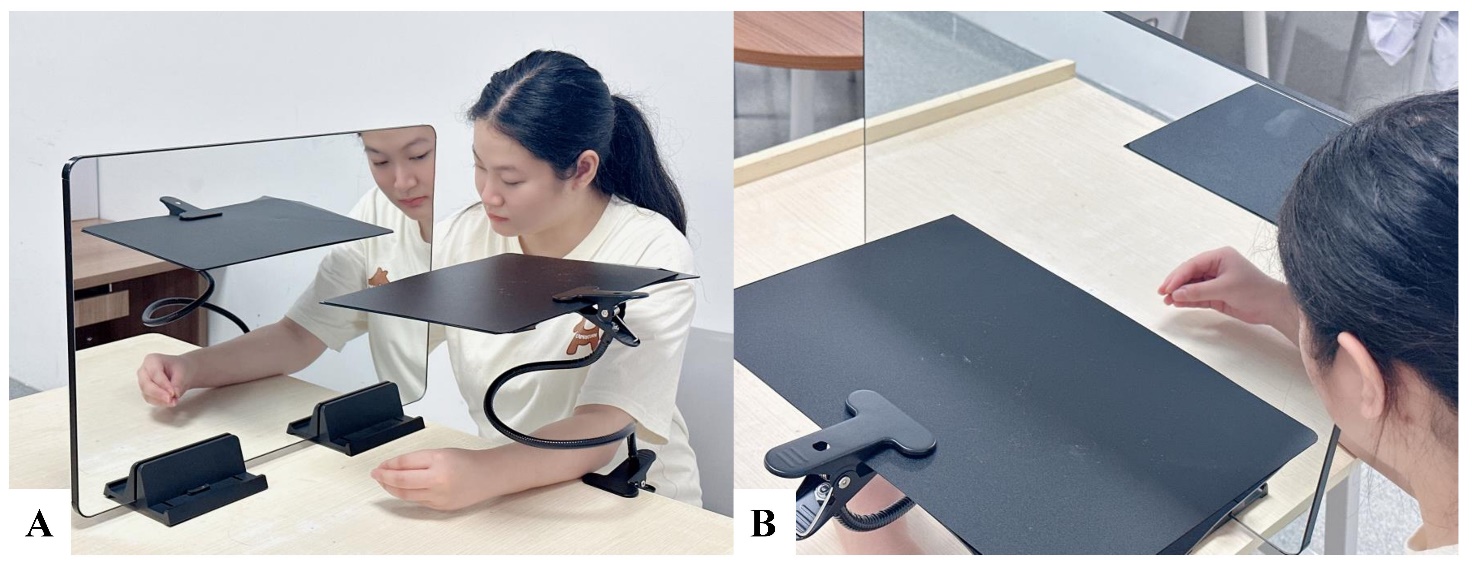


Figure S1 A: the mirror setup of the study; B: a shield used to block the visual input from left side.
